# Supplementary material for: Diploid Male Gametes Circumvent Hybrid Sterility Between Asian and African Rice Species
Source: Front Plant Sci. 2020 Nov 5;11:579305. doi: 10.3389/fpls.2020.579305 (PMC7674174; doi:10.3389/fpls.2020.579305)
Supplement: Supplementary Figure 1 — Information of chromosomal locations (above) and primers (below) for the genotyped loci. [file Data_Sheet_1.PDF]

Supplementary Table S1 Numbers of the induced calli and regenerated plants by AC from the interspecific rice hybrids and their parents

| Rice strains | | Induction medium | No. of cultured anthers | No. of anthers producing callus (%) | No. of replanted calli[a] | No. of regenerated calli (%) |
|---|---|---|---|---|---|---|
| Nipponbare | *O. sativa* | RI-13 | 2,565 | 112 (4.37) | 10 | 0 |
| | | SK-1 | 330 | 23 (6.97) | 23 | 13 (56.52) |
| WK21 | *O. glaberrima* | RI-13 | 13,239 | 146 (1.10) | 10 | 0 |
| Nipponbare × WK21 | F1 | RI-13 | 14,724 | 11 (0.07) | 11 | 1 (9.09) |
| WK21 × Nipponbare | | RI-13 | 13,457 | 87 (0.65) | 87 | 19 (21.84) |

[a] N6-based media were used for inducing plant regeneration.

Supplementary Table S2 Genotypes of the 19 regenerated plants from the calli of WK21/Nip F1 hybrids

| Marker | Nip | WK21 | F1 | #60 | #96 | #13 | #16 | #19 | #20 | #25 | #28 | #47 | #63 | #70 | #74 | #80 | #91 | #38 | #39 | #61 | #79 | #88 |
|---|---|---|---|---|---|---|---|---|---|---|---|---|---|---|---|---|---|---|---|---|---|---|
| 1-1 | N | W | H | N | N | H | H | H | H | H | H | H | H | H | W | H | H | H | H | H | H | H |
| 1-2 | N | W | H | N | N | H | H | W | W | H | W | W | H | H | H | N | H | H | H | H | H | H |
| 2-1 | N | W | H | N | N | H | W | - | H | H | N | H | H | W | H | H | N | H | H | H | H | H |
| 3-1 | N | W | H | W | N | H | W | W | H | N | H | H | H | H | H | W | W | H | H | H | H | H |
| 4-1 | N | W | H | N | N | H | N | H | H | H | W | H | H | W | H | H | H | H | H | H | H | H |
| 4-2 | N | W | H | N | W | H | H | N | H | H | H | N | H | H | H | H | N | H | H | H | H | H |
| 5-1 | N | W | H | W | W | N | H | H | H | H | H | H | N | H | H | H | H | H | H | H | H | H |
| 5-2 | N | W | H | N | W | H | H | W | H | H | H | H | H | H | H | N | H | H | - | H | H | H |
| 6-1 | N | W | H | W | W | H | H | H | H | H | H | H | H | W | H | H | N | H | H | H | H | H |
| 6-2 | N | W | H | N | W | H | H | N | W | H | H | H | W | H | H | H | H | H | H | H | H | H |
| 7-1 | N | W | H | W | N | H | H | H | H | H | H | N | W | H | N | H | H | H | H | H | H | H |
| 7-2 | N | W | H | N | N | H | H | H | H | H | W | H | N | W | N | H | N | H | H | H | H | H |
| 8-1 | N | W | H | N | W | H | H | H | N | H | H | W | N | H | H | W | H | H | H | H | H | H |
| 8-2 | N | W | H | W | W | H | W | H | H | W | N | W | H | N | H | N | H | H | H | H | H | H |
| 9-1 | N | W | H | N | N | N | W | H | H | W | H | W | N | N | H | N | H | H | H | H | H | H |
| 9-2 | N | W | H | N | N | N | H | H | W | H | H | H | H | H | H | H | H | H | H | H | H | H |
| 10-1 | N | W | H | N | W | H | H | W | H | H | H | H | H | H | H | N | H | H | H | H | H | H |
| 10-2 | N | W | H | N | N | H | N | H | H | N | H | H | H | H | H | H | H | H | H | H | H | H |
| 11-1 | N | W | H | N | W | H | H | W | N | W | H | W | H | H | H | H | H | H | H | H | H | H |
| 11-2 | N | W | H | W | N | N | N | H | H | H | N | H | H | H | H | H | W | H | H | H | H | H |
| 12-1 | N | W | H | W | W | H | N | H | H | H | W | H | H | H | H | H | W | H | H | H | H | H |
| 12-2 | N | W | H | N | N | H | N | H | H | W | H | H | W | W | H | H | N | H | H | H | H | H |

A total of 22 markers used here are selected one or two from each of 12 chromosomes and listed in Supplementary Figure S1. The two regenerated plants, #60 and #96, indicated by all the markers were homozygotes (Hom). The next 12 regenerated plants retained both homozygous and heterozygous regions (Hom/Het). The five plants from the right side were judged as heterozygotes (Het). "N", "W", and "H" in the table show homozygous of *O. sativa* (Nip) allele, homozygous of *O. glaberrima* (WK21) allele, and their heterozygous allele, respectively.

Supplementary Table S3 Genetic zygosities of centromeric regions of the 12 chromosomes of 12 regenerated plants and detection of FDR and SDR

| Regen. plant ID | Centromere markers | | | | | | | | | | | | | | | | | | | | | | | | |
|---|---|---|---|---|---|---|---|---|---|---|---|---|---|---|---|---|---|---|---|---|---|---|---|---|---|
| | 1-c1 | 1-c2 | 2-c1 | 2-c2 | 3-c1 | 3-c2 | 4-c1 | 4-c2 | 5-c1 | 5-c2 | 6-c1 | 6-c2 | 7-c1 | 7-c2 | 8-c1 | 8-c2 | 9-c1 | 9-c2 | 10-c1 | 10-c2 | 11-c1 | 11-c2 | 12-c1 | 12-c2 | |
| #13 | H | H | H | H | H | H | H | H | H | H | H | H | H | H | H | H | H | H | H | H | H | H | H | H | FDR |
| #19 | H | H | H | H | H | H | H | H | H | H | H | H | H | H | H | H | H | H | H | H | H | H | H | H | |
| #20 | H | H | H | H | H | H | H | H | H | H | H | H | H | H | H | H | H | H | H | H | H | H | H | H | |
| #25 | H | H | H | - | H | H | H | H | H | H | H | H | H | H | H | H | H | H | H | H | H | H | H | H | |
| #47 | H | H | H | H | H | H | H | H | H | H | H | H | H | H | H | H | H | H | H | H | H | H | H | H | |
| #63 | H | H | H | H | H | H | H | H | H | H | H | H | H | H | H | H | H | H | H | H | H | H | H | H | |
| #74 | H | H | H | H | H | H | H | H | H | H | H | H | H | H | H | H | H | H | H | H | H | H | H | H | |
| #80 | H | H | H | H | H | H | H | H | H | H | H | H | H | H | H | H | H | H | H | H | H | H | H | H | |
| #16 | W | W | W | W | W | W | N | N | W | W | N | N | N | N | W | W | W | W | W | W | N | N | N | N | SDR |
| #28 | W | W | N | N | N | N | W | W | N | N | N | N | W | W | N | N | W | W | W | W | N | N | W | W | |
| #70 | W | W | W | W | W | W | W | W | W | W | W | W | W | W | N | N | N | N | W | W | N | N | W | W | |
| #91 | N | N | N | N | W | W | N | N | N | N | N | N | N | N | N | N | W | W | N | N | W | W | N | N | |

A total of 24 markers in centromeric regions used in this analysis are detailed in Supplementary Figure S1. The 12 regenerated Hom/Het plants retaining both homozygous and heterozygous regions constitute two groups, FDR and SDR.

Supplementary Table S4 Numbers of PMCs indicating normal and anomaly in meiosis I (prophase I) and meiosis II (anaphase II to telophase II)

| | Meiosis I (Diplotene-Diakinesis) | | Meiosis II (Anaphase II-TelophaseII) | |
|---|---|---|---|---|
| | Normal | Anomaly | Normal | Anomaly |
| Nip | 22 | 0 | n.e. | n.e. |
| WK21 | 8 | 0 | 8 | 0 |
| WK21/Nip | 16 | 4 | 22 | 3 |

Anomalies detected in meiosis I and II corresponded with FDR and SDR, respectively. n.e. indicates no examined samples.

Supplementary Table S5  Genotyping of the 12 *HS* loci in the 17 regenerated plants

| *HS* locus | #13 | #19 | #63 | #70 | #91 | #39 | #79 | #88 | #16 | #20 | #25 | #28 | #38 | #47 | #74 | #80 | #96 |
|---|---|---|---|---|---|---|---|---|---|---|---|---|---|---|---|---|---|
| $S_{1}$ | H | H | H | W | H | H | H | H | H | H | H | H | H | H | H | H | W |
| $S_{3}$ | H | H | H | N | W | H | H | H | N | H | H | N | H | W | H | H | W |
| $S_{18}$ | H | H | H | W | N | H | H | H | H | H | H | W | H | H | H | N | W |
| $S_{19}$ | H | H | H | N | W | H | H | H | H | H | H | H | H | H | N | W | W |
| $S_{20}$ | H | H | W | H | N | H | H | H | H | H | H | H | H | H | N | H | N |
| $S_{21}$ | H | H | N | W | N | H | H | H | H | N | W | H | H | H | N | H | N |
| $S_{29}$ *(t)* | H | H | H | H | H | H | H | H | H | H | H | H | H | H | N | N | W |
| $S_{34}$ *(t)* | H | W | N | H | W | H | H | H | W | H | N | H | H | H | H | H | N |
| $S_{36}$ *(t)* | H | H | H | H | H | H | H | H | H | H | H | H | H | H | H | H | W |
| $S_{37}$ *(t)* | H | H | H | W | N | H | H | H | W | H | H | W | H | H | H | H | N |
| $S_{38}$ *(t)* | H | H | H | W | H | H | H | H | N | N | H | W | H | H | H | H | N |
| $S_{39}$ *(t)* | H | N | H | H | N | H | H | H | H | N | N | H | H | N | H | N | N |

The details of markers used in this analysis are listed in Supplementary Figure S1. Except for a Hom plant #96, the 12 Hom/Het plants and five Het plants possess the *HS* loci as partly and completely heterozygous states in the genomes. "N", "W", and "H" in the table show homozygous of *O. sativa* (Nip) allele, homozygous of *O. glaberrima* (WK21) allele, and their heterozygous allele, respectively.

Supplementary Table S6 Pollen fertility, seed set rate, and genotypes of HS loci for the self-pollinated progenies of the three fertile tetraploid lines, RP2-25, RP2-38, and RP2-80

| Plant line | RP2-25 (n=4) | | | | RP2-38 (n=6) | | | | | | RP2-80 (n=5) | | | | |
|---|---|---|---|---|---|---|---|---|---|---|---|---|---|---|---|
| Individual No. | 1 | 2 | 3 | 4 | 1 | 2 | 3 | 4 | 5 | 6 | 1 | 2 | 3 | 4 | 5 |
| Pollen fertility (%) | 4.15 | 4.10 | 6.60 | 8.33 | 0 | 15.44 | 19.84 | 0.86 | 4.39 | 58.30 | 47.06 | n.d. | 7.06 | 37.93 | 64.11 |
| Seed set rate (%)<br>(Fertile / total seeds) | 0<br>(0/44) | 0.54<br>(1/185) | 0<br>(0/86) | 0<br>(0/111) | 0<br>(0/54) | 0<br>(0/42) | 7.92<br>(8/101) | 0<br>(0/44) | 0<br>(0/85) | 39.83<br>(49/123) | 0<br>(0/44) | 0<br>(0/96) | 0<br>(0/13) | 3.23<br>(1/31) | 0<br>(0/38) |
| *HS* locus | Genotypes of RP2 individuals | | | | | | | | | | | | | | |
| *S1* | H | H | H | H | H | H | H | H | H | H | H | H | H | H | H |
| *S18* | H | H | H | H | H | H | H | H | H | H | N | N | N | N | N |
| *S19* | H | H | H | H | H | H | H | H | H | H | W | W | W | W | W |
| *S20* | H | H | H | W | H | H | H | H | W | H | H | H | H | H | H |
| *S21* | W | W | W | W | H | H | H | H | H | H | H | H | H | N | H |
| *S34(t)* | N | N | N | N | H | H | H | H | H | W | - | H | H | - | - |
| *S36(t)* | H | H | H | H | H | H | H | H | H | H | H | H | H | H | H |
| *S37(t)* | H | H | H | H | H | H | H | H | H | H | H | H | H | H | H |
| *S38(t)* | H | H | H | H | H | H | H | H | H | H | H | H | H | H | H |
| *S39(t)* | N | N | N | N | H | H | H | H | H | H | N | N | N | N | N |

"N", "W", and "H" in the table show homozygous of *O. sativa* (Nip) allele, homozygous of *O. glaberrima* (WK21) allele, and their heterozygous allele, respectively.

A

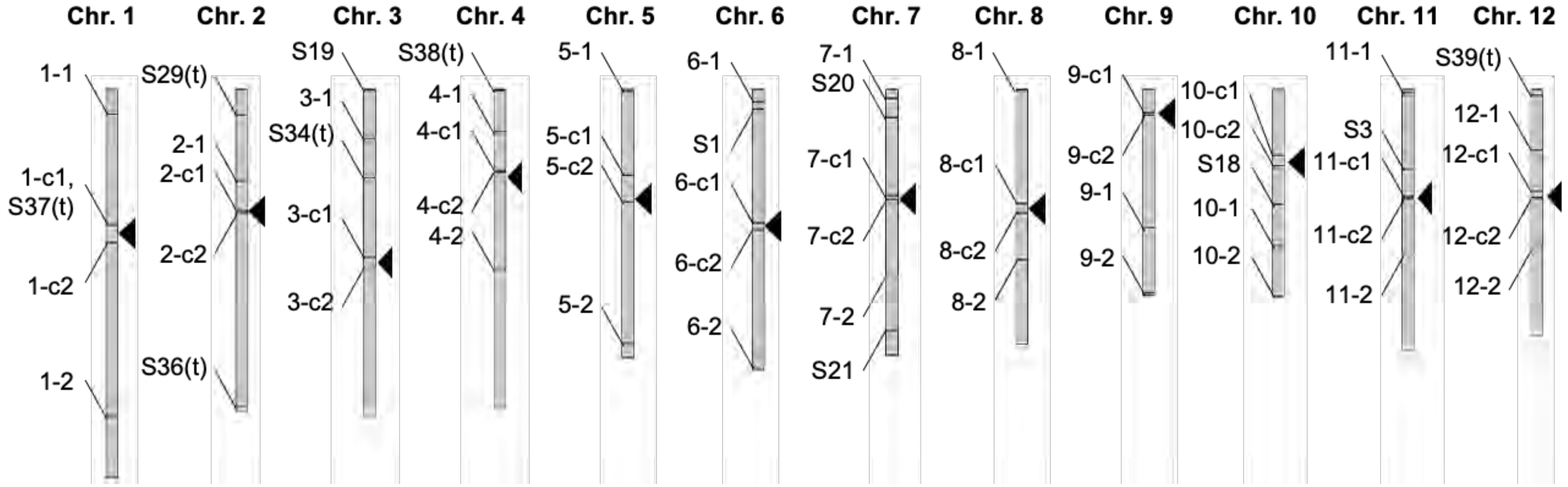


B

| Code | Location (Mbp) | Chr | Primer | Forward sequence | Reverce sequence |
|---|---|---|---|---|---|
| 1-1 | 2.8 | 1 | RM3740 | 5'-ATCCCAACTCTAAGCCACCC-3' | 5'-CTACCCGTCACCAACTCACC-3' |
| S37(t) | 15.1 | 1 | RM449 | 5'-TTGGGAGGTGTTGATAAGGC-3' | 5'-ACCACCAGCGTCTCTCTCTC-3' |
| 1-c1 | 15.1 | 1 | RM449 | 5'-TTGGGAGGTGTTGATAAGGC-3' | 5'-ACCACCAGCGTCTCTCTCTC-3' |
| 1-c2 | 17.1 | 1 | UH1-d2 | 5'-AGGTTTAGCGTCAGTACTTGGG-3' | 5'-ACACGGTTGAATGATAGACACT-3' |
| 1-2 | 36.5 | 1 | RM3825 | 5'-AAAGCCCCCAAAAGCAGTAC-3' | 5'-GTGAAACTCTGGGGTGTTCG-3' |
| S29(t) | 2.9 | 2 | RM279 | 5'-GCGGGAGAGGGATCTCCT-3' | 5'-GGCTAGGAGTTAACCTCGCG-3' |
| 2-1 | 10.2 | 2 | RM3501 | 5'-TCCTAGTGCATCAGCACAGC-3' | 5'-GTCCGTTTCAGCAAGCAAAC-3' |
| 2-c1 | 13.5 | 2 | UH2-u4 | 5'-AGTTAAAAGTTAACTCGCCGACG-3' | 5'-CAGTAAGCATCCTACGGCTCC-3' |
| 2-c2 | 13.8 | 2 | UH2-d1 | 5'-TCAGTGCTCCAGTTCAGCAT-3' | 5'-GGCTGAGGAACCAGGAGTTT-3' |
| S36(t) | 35.4 | 2 | RM207 | 5'-CCATTCGTGAGAAGATCTGA-3' | 5'-CACCTCATCCTCGTAACGCC-3' |
| S19 | 0.1 | 3 | RM60 | 5'-AGTCCCATGTTCCACTTCCG-3' | 5'-ATGGCTACTGCCTGTACTAC-3' |
| 3-1 | 5.5 | 3 | RM5442 | 5'-AGGAGACAGGAAAGCCTTCC-3' | 5'-CGAGTCGACCAGGCTAGAAC-3' |
| S34(t) | 9.8 | 3 | RM7 | 5'-TTCGCCATGAAGTCTCTCG-3' | 5'-CCTCCCATCATTTCGTTGTT-3' |
| 3-c1 | 18.7 | 3 | RM6146 | 5'-ATCTTCTCCGGTTCTCCCTC-3' | 5'-AAGGAGGGAGGAGGCAGTAG-3' |
| 3-c2 | 19.9 | 3 | UH3-d1 | 5'-TGCTGTGAGAATATGTCCACCA-3' | 5'-AGCAAGGGAAGCTATTTACGGA-3' |
| S38(t) | 0.2 | 4 | RM16260 | 5'-TGCTAGTAGGAGAGGAGCGAAGC-3' | 5'-ACTGTGAGTCGTGTCAGAGTTGG-3' |
| 4-1 | 4.7 | 4 | RM6487 | 5'-AGAAGCTGTAGACGATGGCC-3' | 5'-CTAGACCTCATCCCCTTCCC-3' |
| 4-c1 | 9.1 | 4 | UH4-u1 | 5'-CAGGGAGCTGCACTTCTCAA-3' | 5'-TTCAGGTTGCCTCCACCTTG-3' |
| 4-c2 | 9.3 | 4 | UH4-d1 | 5'-TGTATGGTATTCACCCCCGG-3' | 5'-TGGAGCTCTGAGAAATACGAATCC-3' |
| 4-2 | 20.1 | 4 | RM3643 | 5'-AGCATGAGCAGGTGCTAGTG-3' | 5'-CGTTGCATGTGTGATGGC-3' |
| 5-1 | 0.3 | 5 | RM5816 | 5'-CTGCATGCATGACTGCATC-3' | 5'-TGACTCTGATCGATCCATGG-3' |
| 5-c1 | 9.6 | 5 | RM3381 | 5'-ACAAGCACCAGCCACAATAG-3' | 5'-GGTGTTGTTTTGGACGAACG-3' |
| 5-c2 | 12.6 | 5 | UH5-d1 | 5'-TGTGATGTGCAATTGCTCGTC-3' | 5'-TCCAATGGTCCGTTCAATCGT-3' |
| 5-2 | 28.3 | 5 | RM6313 | 5'-ATCCAGATCCACTTTGACCG-3' | 5'-GGAGGACTTCTACCATCCTTG-3' |
| 6-1 | 1.4 | 6 | RM8074 | 5'-TACTACCACTTCTAGATGAGTTCAG-3' | 5'-CTGAATACACTTCAATTTCTCTC-3' |
| S1 | 2.2 | 6 | RM19359 | 5'-GATCCAAGTAGGCACGAGTCACC-3' | 5'-AGCTGATTCCTCTCTGCTTCTCG-3' |
| 6-c1 | 14.9 | 6 | UH6-u2 | 5'-ACCAGTTTTTCCATCTCCAAACC-3' | 5'-CGGCCGGCTAGAAACAAGAT-3' |
| 6-c2 | 15.6 | 6 | UH6-d1 | 5'-ATACCAACACCCGACCACCA-3' | 5'-TTTAGCGGTGGAAGACGACG-3' |
| 6-2 | 31.1 | 6 | RM1031 | 5'-GTGAAGGCACACCAACCG-3' | 5'-GACGAGGATCGAATTCGAAG-3' |

| Code | Location (Mbp) | Chr | Primer | Forward sequence | Reverce sequence |
|---|---|---|---|---|---|
| 7-1 | 1.0 | 7 | RM6222 | 5'-CCTCCTATCCGGAGAAGGAC-3' | 5'-AACACGTTTGAGTTCCAGGC-3' |
| S20 | 3.1 | 7 | RM82 | 5'-TGCTTCTTGTCAATTCGCC-3' | 5'-CGACTCGTGGAGGTACGG-3' |
| 7-c1 | 11.9 | 7 | UH7-u2 | 5'-TCACCGCGGAGAATCAACAA-3' | 5'-AGCCATACCAGCTGATGTCG-3' |
| 7-c2 | 12.3 | 7 | UH7-d1 | 5'-CAGATTCAAACTCTGATACTACCAGG-3' | 5'-TGATCACGTCATATGCTTCAATGTG-3' |
| 7-2 | 20.8 | 7 | RM3826 | 5'-TTAGCTTTCCTCCAGTCTCC-3' | 5'-ACGGGTATCTGAAACACAAC-3' |
| S21 | 26.8 | 7 | RM429 | 5'-TCCCTCCAGCAATGTCTTTC-3' | 5'-CCTTCATCTTGCTTTCCACC-3' |
| 8-1 | 0.1 | 8 | RM6369 | 5'-CAAGCTAGGGCTGCATAAGC-3' | 5'-GCTTCACCTACCTACCTCACC-3' |
| 8-c1 | 12.7 | 8 | UH8-u1 | 5'-TTGGGGGCATAGCTCTTGTT-3' | 5'-GCTGTTCCCCATTTTACTGCA-3' |
| 8-c2 | 13.7 | 8 | UH8-d1 | 5'-TGATCATCACAAGTGTCTTTTCGT-3' | 5'-TCCTCATCGGTCAAAGTGCT-3' |
| 8-2 | 19.0 | 8 | RM3153 | 5'-CGGTTCTTTTCACATGGTCG-3' | 5'-ATCACAACAAGCTCGACGTG-3' |
| 9-c1 | 2.6 | 9 | UH9-u1 | 5'-AACGGACCGATGGTATAGCT-3' | 5'-ATGCTGAGGTAGATTATATGGTGT-3' |
| 9-c2 | 2.9 | 9 | UH9-d1 | 5'-GTGACAGCCAAGTCCGTCAT-3' | 5'-GGTCAAGAAGGTGCAGGTGA-3' |
| 9-1 | 15.4 | 9 | RM3700 | 5'-AAATGCCCCATGCACAAC-3' | 5'-TTGTCAGATTGTCACCAGGG-3' |
| 9-2 | 22.7 | 9 | RM205 | 5'-CTGGTTCTGTATGGGAGCAG-3' | 5'-CTGGCCCTTCACGTTTCAGTG-3' |
| 10-c1 | 7.4 | 10 | C51124 | 5'-CACTTCAGTGCTGGGTGTGC-3' | 5'-TCAAAGGGCAAGTTAACGAC-3' |
| 10-c2 | 8.5 | 10 | UH10-d3 | 5'-ACCGAGCACTTATCTACTTGCA-3' | 5'-GGGGATGATTTCACCCGTAG-3' |
| S18 | 12.8 | 10 | RM25321 | 5'-CACTTCTTGTCACTGTACACACTCC-3' | 5'-CACATGCCGTATATCAACTACCG-3' |
| 10-1 | 17.5 | 10 | RM5620 | 5'-TCGACTTGAAGCATCACACC-3' | 5'-TCTGAAATGTCAAGTGGGCC-3' |
| 10-2 | 23.1 | 10 | RM4771 | 5'-ACGTTGATTTCATTCAGGTC-3' | 5'-ACGCTAACTGAGAAACATGG-3' |
| 11-1 | 0.4 | 11 | RM286 | 5'-GGCTTCATCTTTGGCGAC-3' | 5'-CCGGATTCACGAGATAAACTC-3' |
| S3 | 9.0 | 11 | RM536 | 5'-TCTCTCCTCTTGTTTGGCTC-3' | 5'-ACACACCAACACGACCACAC-3' |
| 11-c1 | 12.0 | 11 | UH11-u1 | 5'-TATCTTTACTGTTACAACGCACCG-3' | 5'-CCATCTCACGGGTGGGTG-3' |
| 11-c2 | 12.2 | 11 | UH11-d1 | 5'-GCTGAAAGTGAAATGTATTGCACC-3' | 5'-ACTAGAACGATACTATAACCCGGT-3' |
| 11-2 | 18.3 | 11 | RM209 | 5'-ATATGAGTTGCTGTCGTGCG-3' | 5'-CAACTTGCATCCTCCCCTCC-3' |
| S39(t) | 0.7 | 12 | RM5568 | 5'-ATTATTGCTTGCGCCTTTAG-3' | 5'-AACGGAACAGATCCAAATG-3' |
| 12-1 | 6.8 | 12 | RM7003 | 5'-GGCAGACATACAGCTTATAGGC-3' | 5'-TGCAAATGAACCCCTCTAGC-3' |
| 12-c1 | 11.4 | 12 | UH12-u3 | 5'-GATTAGGACGGCGTTCTGCT-3' | 5'-AGAAAGGGCTAAACAGGTTCCT-3' |
| 12-c2 | 12.1 | 12 | UH12-d1 | 5'-TGATAGAGCCATGGTGACCG-3' | 5'-TGTTGCAAATATTTTGAAGCGATAAG-3' |
| 12-2 | 17.5 | 12 | RM1261 | 5'-GTCCATGCCCAAGACACAAC-3' | 5'-GTTACATCATGGGTGACCCC-3' |

Supplementary Fig. S1: Information of chromosomal locations (above) and primers (below) for the genotyped loci.
(A) Chromosomal locations of the markers. The markers used for the whole genome zygosities, centromere makers, and *HS* loci markers are indicated by 1-1~12-2 (22 positions), 1-c1~12-c2 (24 positions), and $S_1$~$S_{39}(t)$ (12 loci).
(B) Each marker information includes location in the chromosome, marker name, primer combinations name, and primer sequences.

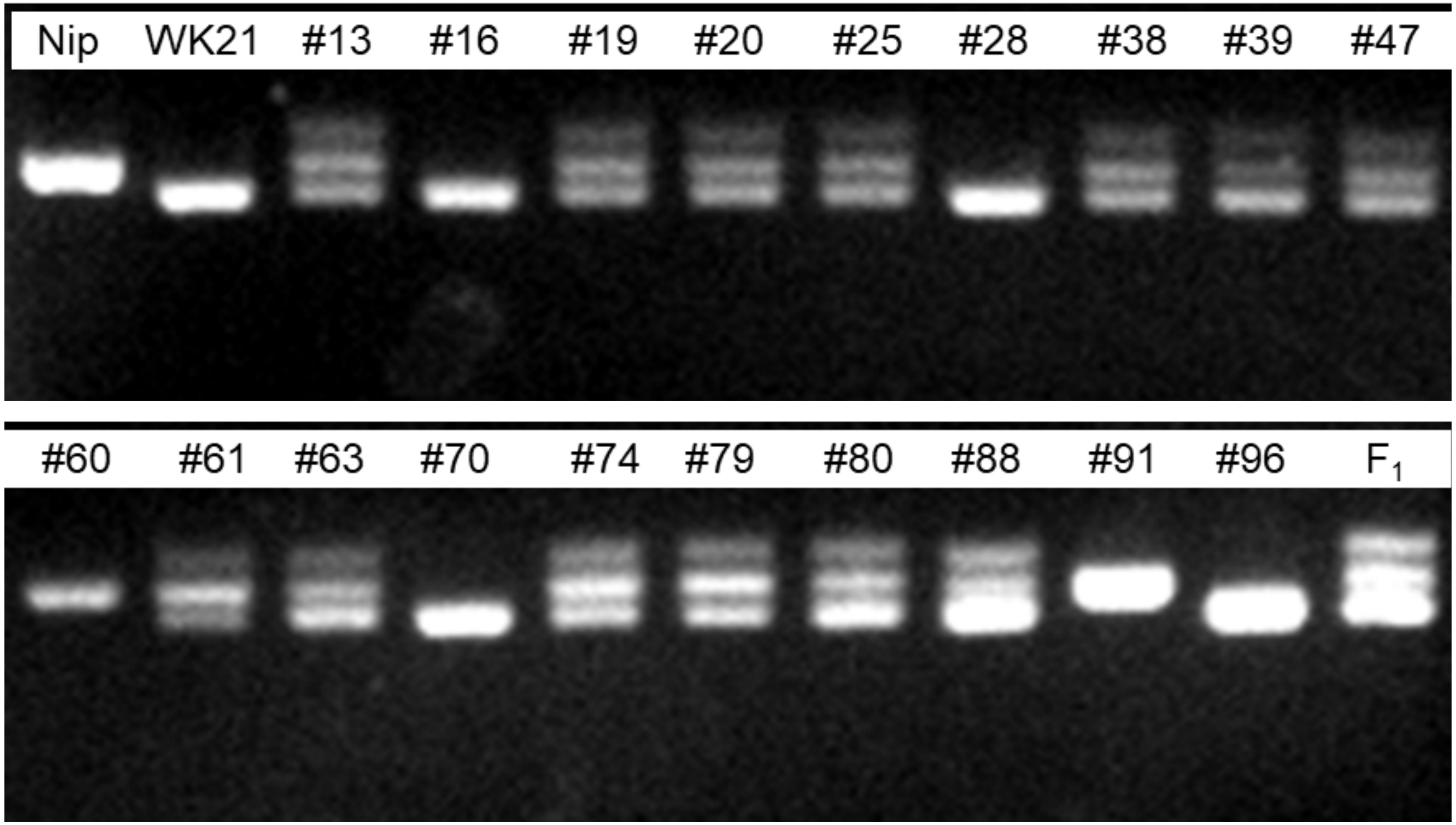


Supplementary Fig. S2: A genotyping profile of the 19 regenerated plants using 10-C2 located on centromere in the chromosome 10.

The gel patterns indicated that the regenerated plants, #60 and #91 possessed Nip homozygous allele, #16, #28, #70, and #96 retained WK21 homozygous allele. FDR gametes: #13, #19, #20, #25, #47, #63, #74, #80, and #88 had heterozygous alleles at 10-c2. SDR gametes: #16, #28, #70, and #91.

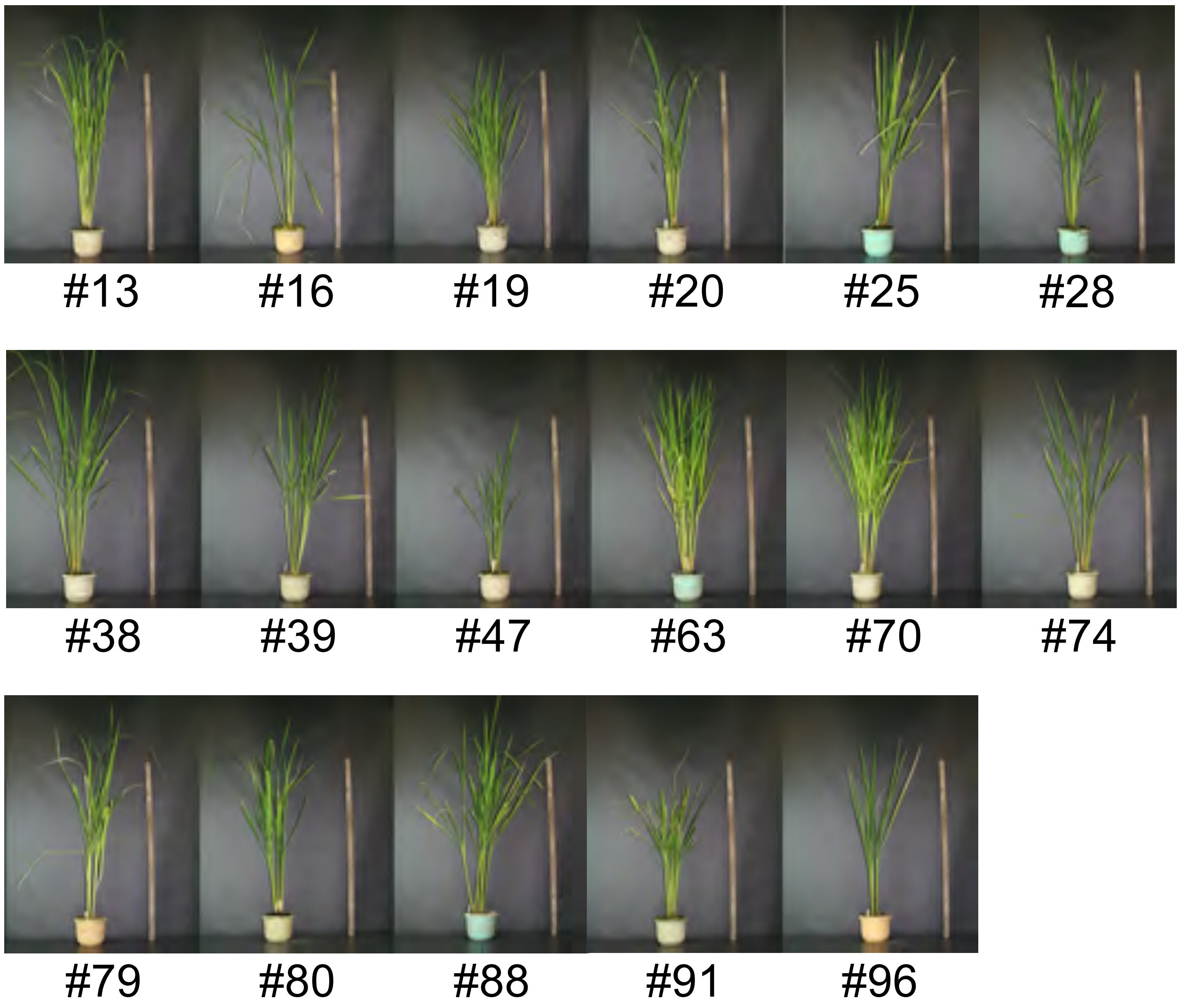


Supplementary Fig. S3: Seventeen regenerated plants obtained from AC of WK21/ Nip $F_1$. These 17 regenerants exhibited different shapes. Initially, we obtained 19 regenerants, but two regenerants, #60 and #61, died after transplantation to soil, so DNA was isolated from the two plants.
